# CRISPR/Cas9-mediated disruption of *MdGA20ox* confers dwarfism in apple

**DOI:** 10.1093/hr/uhag083

**Published:** 2026-03-04

**Authors:** Nan Guo, Ningwang Huang, Yao Xiong, Doudou Chen, Haochen Sun, Qian Yang, Haorui Zhang, Yi Wang, Zhenhai Han, Wei Li

**Affiliations:** College of Horticulture, China Agricultural University, Beijing 100193, China; College of Horticulture, China Agricultural University, Beijing 100193, China; College of Horticulture, China Agricultural University, Beijing 100193, China; College of Horticulture, China Agricultural University, Beijing 100193, China; College of Horticulture, China Agricultural University, Beijing 100193, China; College of Horticulture, China Agricultural University, Beijing 100193, China; College of Horticulture, China Agricultural University, Beijing 100193, China; College of Horticulture, China Agricultural University, Beijing 100193, China; College of Horticulture, China Agricultural University, Beijing 100193, China; College of Horticulture, China Agricultural University, Beijing 100193, China

Dear Editor,

Apple (*Malus domestica*) is a major fruit crop worldwide and holds considerable economic importance in China. In modern orchard systems, dwarfing and high-density planting have emerged as dominant strategies. The extensive adoption of dwarf rootstocks has notably increased orchard productivity and profitability by reducing tree height, simplifying crop management, enhancing photosynthetic efficiency, optimizing land use, and promoting earlier flowering and fruit production [1].

Gibberellins (GAs) were particularly critical, governing various developmental stages including seed germination, root and stem growth, floral initiation, and internode elongation. GA20-oxidase (GA20ox) is a key enzyme in GA biosynthesis [2]. CRISPR/Cas9 technology has been successfully applied in various crops to improve agronomic traits. In banana, CRISPR/Cas9-mediated editing of *MaGA20ox2* has led to a semi-dwarf phenotype [3]. This study employed CRISPR/Cas9 technology to edit the *MdGA20ox* gene in apple, which induced a stable dwarf phenotype, whereas its overexpression promoted plant growth.

To assess its functional role in regulating plant height, the 1179 bp coding sequence of *MdGA20ox* was cloned into an overexpression vector and transformed into tobacco (*Nicotiana tabacum*). Transgenic tobacco plants overexpressing *MdGA20ox* exhibited a marked increase in plant height, internode length, and internode number compared to wild-type (WT) controls. Specifically, transgenic plants were 2.3 (OE#1) and 2.5 (OE#14) times as tall as WT, with both internode number and length about 1.5 times those of WT (Fig. 1A and B). These results demonstrated that overexpression of *MdGA20ox* significantly promotes plant growth and internode development. To validate these findings in apple, we generated transgenic GL-3 apple lines overexpressing *MdGA20ox* (Fig. 1C). Quantitative reverse transcription polymerase chain reaction (qRT-PCR) analysis confirmed that *MdGA20ox* transcript levels in OE#2 and OE#9 were 3.8 and 4.1 times as high as those in the WT, respectively. The overexpression of *MdGA20ox* resulted in a significant increase in its expression level (Fig. 1D) and plant height in OE#9 (Fig. 1E).

**Figure 1:**
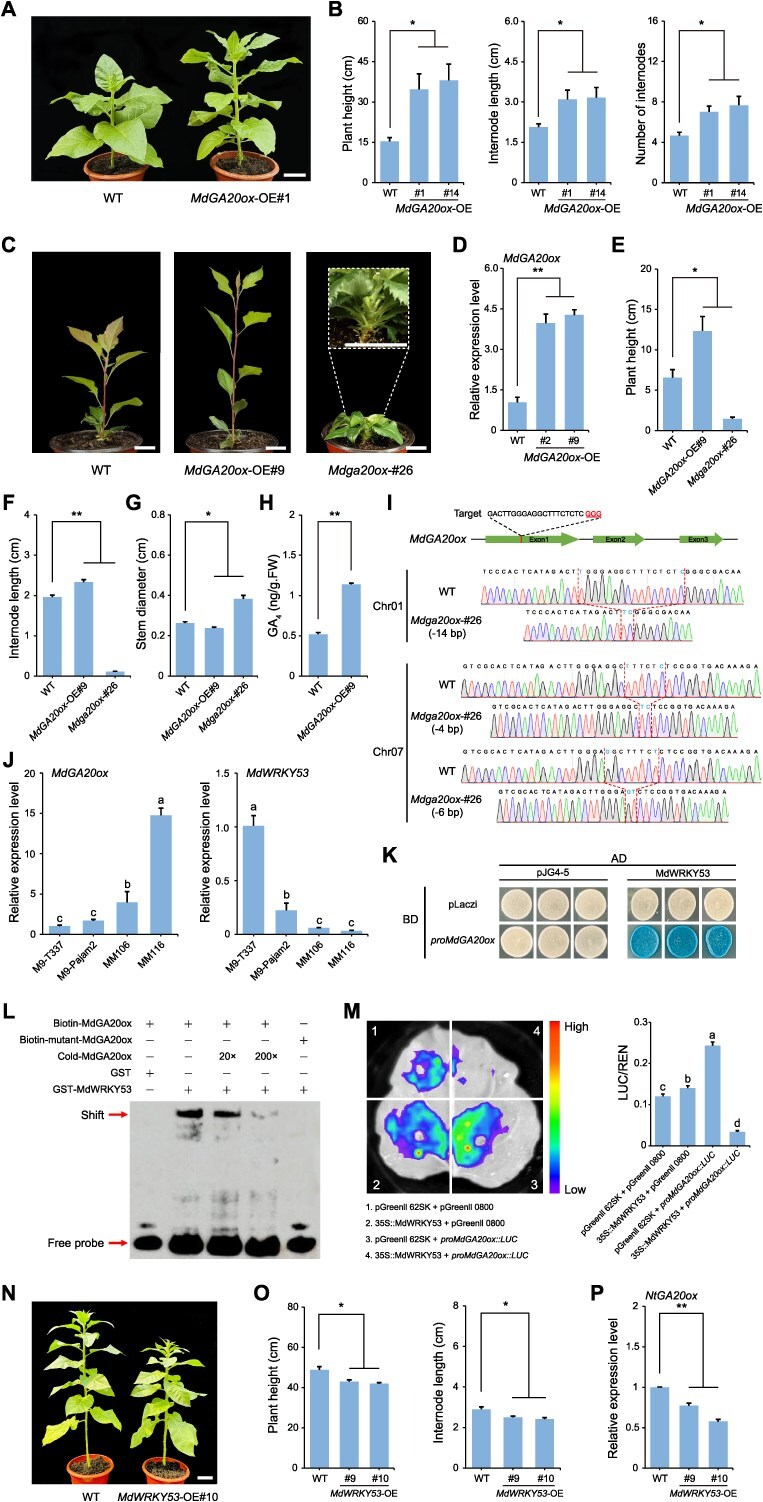
Functional analysis of apple *MdGA20ox*. (**A**) Phenotype of transgenic tobacco lines overexpressing *MdGA20ox* (*MdGA20ox-*OE#1). Scale bar = 5 cm. (**B**) Plant height, internode length, and internode number in *MdGA20ox* transgenic tobacco lines. (**C**) Phenotype of *MdGA20ox* OE#9 and mutant #26 compared to WT. Scale bar = 2 cm. (**D**) Expression levels of *MdGA20ox* in apple lines (*MdGA20ox-*OE#2, OE#9). (**E**) Plant height in *MdGA20ox* OE#9 and mutant #26 compared to WT. (**F**) The internode length of *MdGA20ox* OE#9 and mutant #26 compared to WT. (**G**) The stem diameters of *MdGA20ox* OE#9 and mutant #26 compared to WT. (**H**) GA_4_ levels in *MdGA20ox* OE#9 and wild-type plants were quantified by liquid chromatography–tandem mass spectrometry (LC–MS/MS). (**I**) Design of the target site for *MdGA20ox.* Verification of mutation types in *Mdga20ox* mutant #26 in Chr01 and Chr07 using TA cloning and Sanger sequencing. (**J**) Expression levels of *MdGA20ox* and *MdWRKY53* in different apple rootstocks. (**K**) Yeast one-hybrid assay demonstrating the interaction between MdWRKY53 and the *MdGA20ox* promoter. Three independent yeast colonies were assessed, and results were observed on day 3. (**L**) Electrophoretic mobility shift assay (EMSA) results showing that GST-tagged MdWRKY53 (GST-MdWRKY53) binds to a (5′) biotin-labeled *MdGA20ox* promoter probe. GST alone was used as a negative control. (**M**) Dual-luciferase reporter assay showing that MdWRKY53 represses *MdGA20ox* promoter activity. The color scale indicates luminescence intensity, and the accompanying histogram shows the ratio of firefly luciferase to Renilla luciferase activity. (**N**) Phenotype of a transgenic tobacco line overexpressing *MdWRKY53* (*MdWRKY53-*OE#10). Scale bar = 5 cm. (**O**) Plant height and internode length in *MdWRKY53* transgenic tobacco plants. (**P**) Expression levels of *NtGA20ox* in *MdWRKY53* transgenic tobacco plants (OE#9 and OE#10). Significant differences were determined by Student’s *t*-test (^*^P < 0.05, ^**^P < 0.01). The different lower letters indicate significant differences according to Duncan’s test (*P* < 0.05). The error bars indicate standard errors.

Although CRISPR technology has been extensively used for targeted gene knockout in plants, its application to induce dwarfism through precise gene editing in apple remains underexplored. In apple, two homologous *GA20ox* genes are present, located on chromosomes 01 and 07, respectively. To simultaneously target both genes, the single guide RNA (sgRNA) target site was designed based on conserved sequences within their coding regions. Transgenic lines were initially screened using Cas9-specific primers. Subsequently, the target regions were amplified using site-specific primers, and the resulting PCR products were analyzed by Sanger sequencing. One representative line #26, which exhibited successful editing events, was selected for further analysis. In line #26, the Chr01 contained a homozygous 14-bp deletion, and the Chr07 exhibited biallelic deletions of 4 and 6 bp (Fig. 1I). Phenotypic analysis showed that the plant height of mutant #26 was substantially reduced by 78% compared to the WT (Fig. 1C and E). In addition, comparative analyses of stem diameter and internode length relative to the WT revealed that *MdGA20ox* overexpressing line exhibited a reduced stem diameter and increased internode length, whereas the mutant line showed an increased stem diameter and shortened internodes (Fig. 1F and G). Given that MdGA20ox is a key enzyme in gibberellin biosynthesis, liquid chromatography–tandem mass spectrometry (LC–MS/MS) analysis revealed that the GA_4_ content was significantly higher in the *MdGA20ox* overexpressing line compared with the WT, while GA_4_ content was undetectable in the mutant lines. These results suggested that *MdGA20ox* might regulate plant height by affecting GA_4_ synthesis (Fig. 1H). In summary, these findings demonstrated that *MdGA20ox* plays a critical role in regulating apple plant height, and that targeted mutations can produce stable dwarf phenotypes suitable for high-density orchard systems.

To further study the relationship between *MdGA20ox* expression and apple dwarfing rootstocks, we analyzed *MdGA20ox* transcript levels in different rootstocks. The results showed that *MdGA20ox* expression was significantly lower in dwarfing rootstocks (‘M9-T337’ and ‘M9-Pajam2’) than in ‘MM106’ and ‘MM116’. Promoter analysis of *MdGA20ox* identified a conserved W-box element (C/TTGACC/T), a known binding motif for WRKY transcription factors. Notably, previous studies have demonstrated that overexpression of *WRKY53* in rice reduced plant height [4]. And the expression pattern of *MdWRKY53* in different apple rootstocks is opposite to that of *MdGA20ox*, which suggested that *MdWRKY53* might play an important role in the apple dwarfing process (Fig. 1J). To determine whether MdWRKY53 directly regulates *MdGA20ox*, we performed yeast one-hybrid (Fig. 1K), electrophoretic mobility shift assay (Fig. 1L), and dual-luciferase assays (Fig. 1M). Yeast one-hybrid and EMSA assays confirmed that MdWRKY53 directly binds to the *MdGA20ox* promoter, and dual-luciferase assays further showed that MdWRKY53 significantly reduced *MdGA20ox* promoter-driven luciferase activity. Taken together, these findings indicated that MdWRKY53 functions as a transcriptional repressor of *MdGA20ox* and may play a critical role in the regulation of apple dwarfing. We have generated *MdWRKY53* overexpressing lines in tobacco using *Agrobacterium*-mediated transformation. Two representative transgenic lines (*MdWRKY53*-OE#9, OE#10) were selected for phenotypic analysis. Compared to WT plants, these lines exhibited significant reductions in plant height by 12% and 14%, respectively. Correspondingly, internode lengths were reduced by 14% and 17%, respectively (Fig. 1N and O). Moreover, the expression levels of *NtGA20ox* in *MdWRKY53*-OE lines were substantially reduced by 23% and 42% in OE#9 and OE#10, respectively, compared to WT plants (Fig. 1P). In summary, these findings demonstrate that MdWRKY53 regulates plant dwarfism by repressing the expression of *MdGA20ox*, providing critical insights into the molecular mechanisms underlying plant height regulation in apple.

*Mdga20ox* mutants exhibit a pronounced dwarfing phenotype, which provides potential application value for apple production [5]. Reduced shoot elongation and shortened internodes effectively limit excessive vegetative growth, resulting in compact tree architecture. Such growth restraint is particularly advantageous for high-density planting systems, where precise control of tree size is essential to optimize light interception and spatial arrangement. The moderated vegetative vigor of *Mdga20ox* mutants also reduces the need for intensive pruning practices, thereby lowering labor inputs and enhancing management efficiency in intensive orchards.

In conclusion, this study employed CRISPR/Cas9 technology to edit the *MdGA20ox* gene in apple. Overexpression of *MdGA20ox* promoted plant growth, whereas its knockout induced a stable dwarf phenotype. Furthermore, MdWRKY53 was found to bind to the W-box element in the *MdGA20ox* promoter, repressing its expression and contributing to the dwarf phenotype. These findings provide an important genetic basis for the development of compact-growth germplasm suitable for modern apple production systems.

## Data Availability

All the data generated or analyzed during this study are included in this article.
